# Comparative Study and Real-World Validation of Vertical Load Estimation Techniques for Intelligent Tire Systems

**DOI:** 10.3390/s25072100

**Published:** 2025-03-27

**Authors:** Ti Wu, Xiaolong Zhang, Dong Wang, Weigong Zhang, Deng Pan, Liang Tao

**Affiliations:** 1 School of Instrument Science and Engineering, Southeast University, Nanjing 210096, China; wuti@seu.edu.cn; 2 School of Engineering, Anhui Agricultural University, Hefei 230036, China; xlzhang@ahau.edu.cn (X.Z.); 2024094@tlu.edu.cn (L.T.); 3 Giti Tire (China) R&D Center, No.88 Danxia Road, Hefei 230601, China; pan.deng@giti.com

**Keywords:** vertical load estimation, intelligent tire technology, accelerometer, strain, PVDF sensor, sensor comparison, finite element modeling (FEM), road validation

## Abstract

Accurate vertical load measurement through intelligent tire technology is crucial for vehicle stability, handling, and safety. Existing studies have mainly focused on modeling and bench experiments, overlooking a detailed comparative analysis of real sensor performance and validation under actual driving conditions. This study addresses this gap by performing sensor comparisons and extensive real-road validation to ensure the accuracy and reliability of the proposed methods. First, finite element modeling (FEM) is used to assess the feasibility of accelerometer and strain-based sensors for vertical load prediction. High-precision bench tests quantitatively compare the performance of multiple triaxial Integrated Electronics Piezoelectric (IEPE) accelerometers and Polyvinylidene Fluoride (PVDF) sensors, identifying accelerometers as the superior choice due to their better stability and linearity. Vertical load prediction algorithms are developed using Support Vector Machine (SVM) and linear regression, considering variables like contact length, vehicle speed, and tire pressure. The algorithms are validated under real-road conditions using high-performance instruments across constant speed, acceleration, braking, and cornering, and a self-designed compact Intelligent Tire Test Unit (ITTU) is deployed for product-level implementation, confirming its effectiveness in real-world driving scenarios. The findings provide a validated framework for accurate vertical load estimation and real-time tire parameter prediction, offering practical insights for improving intelligent tire technology in dynamic driving conditions.

## 1. Introduction

Tire vertical load is a critical parameter in vehicle dynamics, influencing key aspects such as traction, braking performance, handling, and the operation of active safety systems [1,2,3]. Accurate vertical load estimation is essential for various applications, including vehicle stability control [1,2], adaptive suspension systems [3], and real-time tire condition monitoring [4]. Traditional estimation approaches primarily rely on vehicle dynamic models [5,6] or external sensors such as wheel force transducers (WFTs) [7,8]. However, these methods often incur high costs, exhibit limited real-time capabilities, and lack direct sensing of tire–road interactions.

Recently, intelligent tire technology has emerged as a promising solution for vertical load measurement [9,10,11] and various tire–road interaction sensing tasks, including contact patch evolution [12,13], tire forces [10,14,15], slip characteristics [16,17,18], tire wear [9,19,20,21], and road conditions [22,23,24,25,26,27].

Among the various sensing methods for intelligent tire technology, accelerometers [9,10,14,16,19,20,21,24,26,27,28,29] and strain-based sensors, including strain gauges [4,12,15,30,31] and PVDF sensors [17,18,25,32], have gained significant attention due to their compact size, cost-effectiveness, and potential for real-time deployment [33]. Installed inside the tire and rotating circumferentially with it, these sensors capture periodic variations in acceleration and strain at specific tread locations, which are then translated into tire behavior patterns through signal correlation analysis [10,18,19,26,28]. During this process, accurately recognizing tire contact characteristics typically serves as an intermediate step for further estimation of tire parameters in many sensing strategies [10,12,27,30,31], which will be given particular attention in the subsequent research.

Despite their potential, intelligent tire technology faces challenges in achieving practical applications. Firstly, existing research on intelligent tire technology has largely focused on model-based analyses (e.g., FEM) [26,34] and bench testing [9,19,29,30,32] to recognize sensor response patterns and develop corresponding prediction algorithms, with limited emphasis on real-road testing. However, FEM alone is insufficient for real-world validation, as it introduces unavoidable errors due to uncertainties in boundary conditions, material parameters, and approximations in geometry and contact modeling [35]. Similarly, while bench testing is valuable for early-stage development by enabling controlled experiments to identify sensor response patterns, it is inherently limited by its simplified and controlled environment. It cannot fully replicate real-world complexities such as dynamic load variations, the influence of the complete vehicle system, and external factors like weather, road irregularities, and noise. Therefore, real-road testing is crucial to evaluate the real-world performance of the developed technology.

Additionally, prior studies have primarily focused on single-sensor methods, with little attention to systematically comparing different sensing technologies for practical applications. However, a comparative study that explores their respective signal characteristics and sensing performance is highly valuable. Such research can facilitate selecting the optimal sensor type for large-scale deployment, achieving enhanced sensing performance through multi-sensor integration and data fusion by leveraging the advantages of different sensing approaches, and improving environmental adaptability by adopting more stable sensors and applying adaptive compensation strategies under their respective reliable conditions.

**Figure 1:**
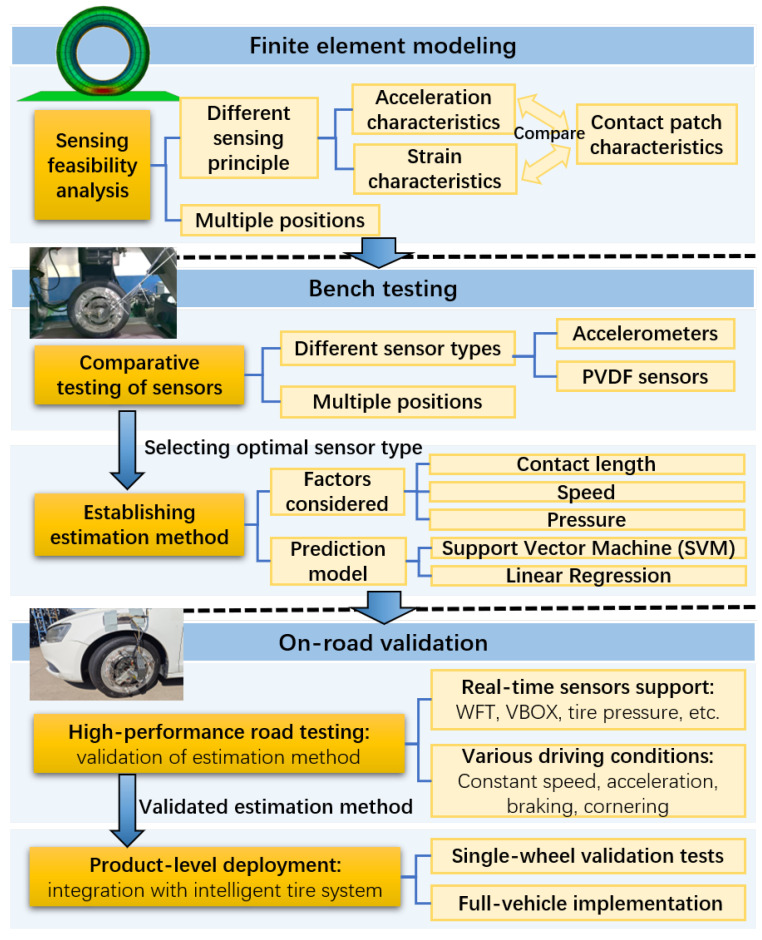
Technical roadmap of the study for vertical load estimation.

To address these gaps, this study focuses on comparing accelerometer-based and strain-based sensing methods and proposes practical solutions for vertical load estimation in real-road applications, supported by FEM theoretical analysis, high-precision bench testing, and real-road validation, as shown in Figure 1. The FEM analysis offers theoretical insights and conceptual guidance on sensor feasibility, which are subsequently validated through controlled bench testing. Finally, real-world road experiments confirm the practical applicability of the developed estimation method. The contributions of this study are as follows:

- **Comparative study of sensing methods:** A comprehensive comparison of accelerometer-based and strain-based sensing methods for vertical load estimation is conducted using finite element analysis (FEA) and high-precision bench testing, demonstrating the superior stability and linearity of the accelerometer-based sensing method.
- **Real-road validation and product-level deployment:** The vertical load estimation method is rigorously validated under dynamic real-road conditions using high-performance instruments across constant speed, acceleration, braking, and cornering. Further product-level testing with the self-designed ITTU, through both single-wheel and full-vehicle implementations, confirms its effectiveness in real-world scenarios, bridging the gap between theoretical models and practical deployment.

The remainder of this paper is organized as follows. Section 2 presents the FEM and feasibility analysis of accelerometer-based and strain-based sensing approaches for vertical load estimation. Section 3 compares the sensor signal characteristics and vertical load estimation accuracy of each sensor type in bench tests, establishing the vertical load prediction method based on the optimal sensor type. Section 4 details the real-road validation and product-level deployment. Section 5 concludes the paper and outlines future research directions.

## 2. Finite Element Modeling and Feasibility Analysis

This section presents the finite element simulation to evaluate the feasibility of acceleration-based and strain-based methods for tire contact analysis and vertical load estimation. Additionally, since some sensors (e.g., PVDF sensors) measure dynamic changes rather than absolute values [36], the derivative of the strain curve is also provided to aid in understanding sensor response characteristics.

### 2.1. Finite Element Modeling of Tire Contact Sensing

**Figure 2:**
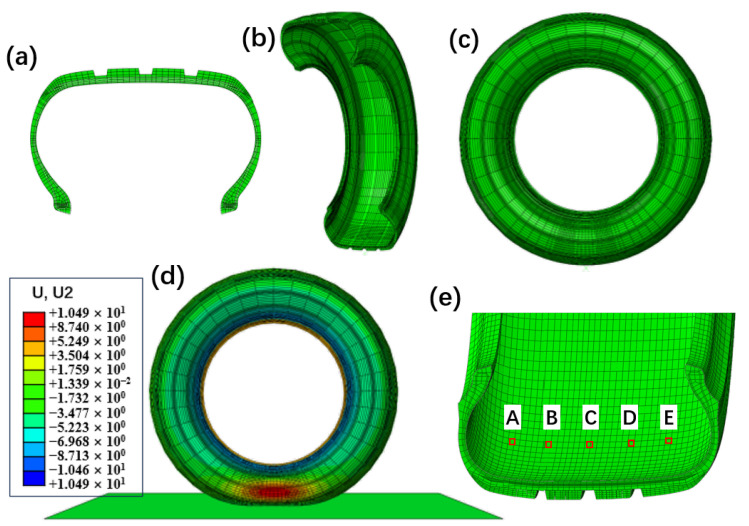
Tire FEM process: (**a**) 2D cross-section tire model, (**b**) partial 3D reconstruction by rotating the cross-section, (**c**) full 3D tire model, (**d**) vertical load deformation diagram of the tire 3D model, (**e**) simulation point locations on the tire’s inner surface, labeled A–E.

A finite element model of a 195/65R15 semi-steel radial tire was developed using Abaqus/CAE to investigate the feasibility of acceleration-based and strain-based sensing methods. The modeling process consisted of two main steps. A 2D axisymmetric cross-sectional model of the tire was first constructed, as shown in Figure 2a. The *SYMMETRIC MODEL GENERATION command in the input file (.inp) was used to revolve the 2D model 360° around the rotational axis, generating the full 3D tire model while preserving the material properties, section definitions, and element sets from the original 2D model (Figure 2b,c).

The rubber material was defined using the Yeoh hyperelastic model [35], which is a special form of a three-term strain energy density function. The Yeoh model was chosen for its ability to accurately capture the nonlinear stress–strain behavior of rubber under large deformations while requiring fewer material parameters than other hyperelastic models. The material parameters were obtained through experimental fitting and are listed in Table 1. The reinforcement layers, including cords in the carcass ply, steel belts, bead wires, and chafer layers, were modeled using the rebar layer operation, and their material properties are given in Table 2. In the finite element model, these reinforcement layers were treated as embedded elements within the rubber matrix, assuming a perfect bond between the reinforcements and the surrounding rubber. This assumption simplifies the material interaction while maintaining the overall structural integrity of the tire model. Constraints were applied to embed the reinforcements within the rubber matrix to ensure accurate material interaction. For element selection, the rubber components in the 2D model were meshed using CGAX3H (triangular) and CGAX4H (quadrilateral) elements, which are hybrid axisymmetric elements suitable for rubber materials. The reinforcement layers were modeled using SFMGAX1 elements, specifically designed for embedded structural reinforcements. After constructing the 3D tire model, a rigid road surface was added to establish the contact and loading conditions, as depicted in Figure 2d. The further details and experimental validation of the tire finite element model can be found in reference [37].

**Figure 3:**
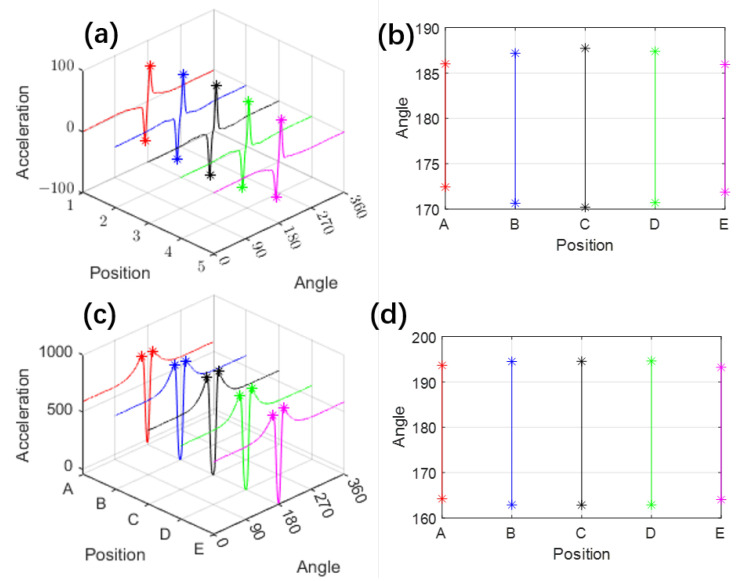
Simulated acceleration results: (**a**) Circumferential acceleration distribution with characteristic points (one valley and one peak); (**b**) Tire rotation angles corresponding to the characteristic points of circumferential acceleration; (**c**) Radial acceleration distribution with characteristic points (two peaks); (**d**) Tire rotation angles corresponding to the characteristic points of radial acceleration. The ‘*’ points denote the selected characteristic points.

**Figure 4:**
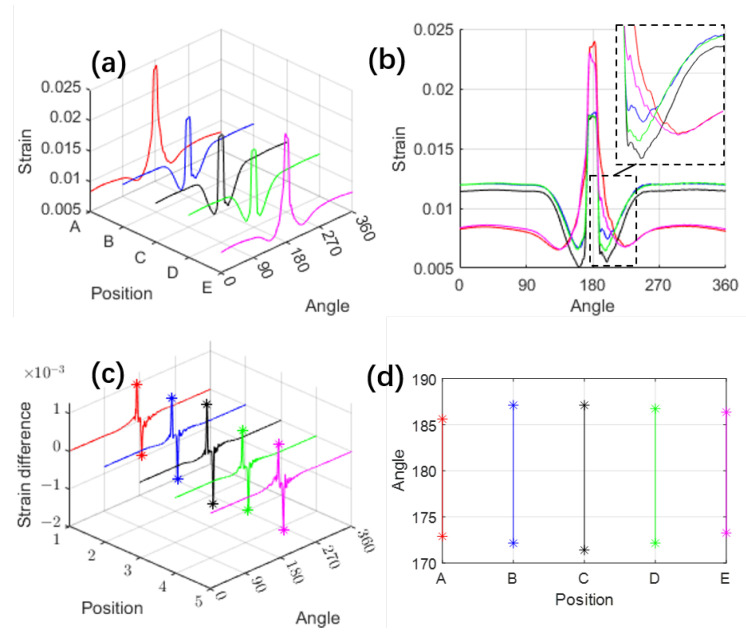
Simulated strain results: (**a**) Circumferential strain distribution; (**b**) Side view of (**a**) projected in the strain-angle plane; (**c**) Circumferential strain variation with characteristic points (one peak and one valley); (**d**) Tire rotation angles corresponding to the characteristic points of circumferential strain variation. The ‘*’ points denote the selected characteristic points.

A steady-state rolling simulation was conducted at a constant speed of 50 km/h, with an inflation pressure of 240 kPa and a vertical load of 4000 N. Five different locations on the inner liner of the tire were selected for analysis, as indicated in Figure 2e, to evaluate their acceleration and strain responses under these conditions. The corresponding acceleration results are presented in Figure 3, while the strain distributions are shown in Figure 4.

**Figure 5:**
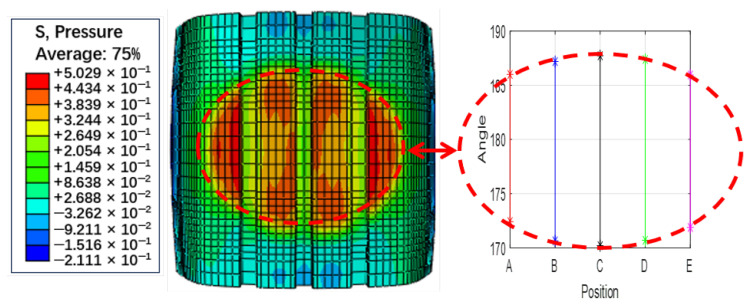
Simulated contact footprint results (contact pressure distribution) and their correlation with sensor characteristics (illustrated using Figure 3b as an example).

To further analyze the sensor response to tire–ground interaction, the contact footprint under the same operating conditions was simulated, as illustrated in Figure 5. These footprint data provide insight into how different sensor types capture ground contact characteristics, aiding in the interpretation of their sensing behavior.

### 2.2. Analysis of FEM Simulation Results

As illustrated in Figure 3, both circumferential and radial acceleration (in the tire coordinate system) exhibit distinct features near the tire–road contact area. For circumferential acceleration, characteristic points can be identified at the peaks and valleys. In contrast, radial acceleration shows a pronounced valley at the contact region, with two smaller peaks selected as characteristic points.

The circumferential strain behavior depicted in Figure 4 also reveals a prominent peak as the tire approaches the contact area. However, upon closer examination (Figure 4b), the valleys on either side of this peak are less smooth compared to those in the radial acceleration, making them less suitable as characteristic points. Therefore, the differentiated strain curve (Figure 4c) is used to identify peaks and valleys as characteristic points for circumferential strain.

These sensors’ characteristic points are projected onto the position-angle plane, as shown in Figure 3b,d and Figure 4d, which illustrate the distribution of sensor characteristic points across the tire surface. To further analyze their relevance, we compare these distributions with the actual tire contact patch (Figure 5). Although the two sets of figures use different measurement units, making their shapes not perfectly aligned, a clear resemblance can still be observed—the envelope formed by these characteristic points closely mirrors the shape of the tire’s contact patch. The rotation angles corresponding to the five simulation elements show a pattern with a longer middle section and shorter ends, forming an elliptical shape.

Due to factors such as tire thickness and the specific sensitivity regions of different sensors, these characteristic points do not always coincide precisely with the exact contact boundaries. For instance, the characteristic points corresponding to circumferential and radial accelerations appear at different locations. However, despite these limitations, the FEM simulation still provides critical insights: the shape enclosed by these characteristic points effectively delineates the contact area, and their distribution exhibits a strong correlation with the contact patch boundaries.

Building upon this observation, we define the angular range (or length) enclosed by these characteristic points as a generalized tire contact length, which can serve as a representation of the contact patch. Given that the contact area is directly related to vertical load, this approach can further support vertical load estimation.

Thus, the FEM analysis provides a qualitative assessment of the feasibility of using internal tire acceleration and strain characteristics for vertical load estimation. The conceptual framework for vertical load prediction involves calculating the contact length from characteristic points such as the peak and valley responses of the sensor signals, and then using the relationship between contact length and vertical load to predict the vertical load.

However, due to the complex nonlinear behavior of tires, inherent discrepancies between FEM simulations and real-world conditions, and sensor-related measurement uncertainties, further refinement of the FEM to precisely map characteristic points to contact boundaries and establish specific prediction models may not accurately reflect the actual sensor performance. Therefore, the focus will shift to rigorous bench testing, where the sensor characteristics will be quantitatively evaluated and validated under real-world conditions to establish their correlation with contact properties and vertical load.

## 3. Bench Test Validation and Comparative Analysis

To evaluate the feasibility and compare the performance of accelerometer-based and strain-based sensing methods, a high-performance bench test was conducted. Multiple triaxial IEPE accelerometers (Model 3333M2T, Dytran Instruments, Inc., CA, USA) and PVDF sensors were mounted inside the tire to analyze their signal characteristics at various in-tire locations and under varying vertical loads, tire pressures, and vehicle speeds, facilitating the assessment of their potential for vertical load estimation.

### 3.1. TestSetup and Methodology

**Figure 6:**
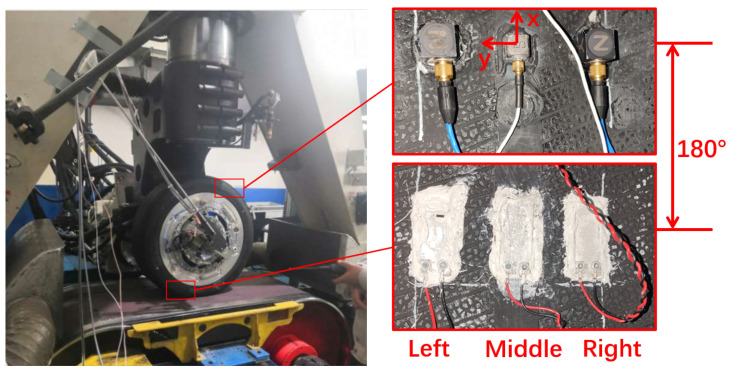
Bench test setup using the MTS Flat-Trac system (**left**), and the in-tire sensors: three accelerometers (**top right**) and three PVDF sensors (**bottom right**) mounted on the inner tire liner.

The experiment was conducted using a Giti 225/45 R17 radial tire on an MTS Flat-Trac system (as shown in Figure 6), which provides a controlled test environment with precise force and motion measurements. Three triaxial accelerometers and three PVDF sensors were mounted on the inner tire liner, positioned 180 degrees apart in the circumferential direction. This configuration enabled a comparative study of their signal characteristics and estimation performance under identical operating conditions.

All sensors were wired to the data acquisition system via a slip ring to ensure stable signal transmission during tire rotation. High-frequency sampling at 50 kHz was used for the accelerometers, PVDF sensors, and an integrated rotation angle sensor. Additionally, an in-tire pressure and temperature sensor (Model PCM-67, EFE, Annecy, France) was installed, providing synchronized 100 Hz sampling to monitor internal conditions.

**Figure 7:**
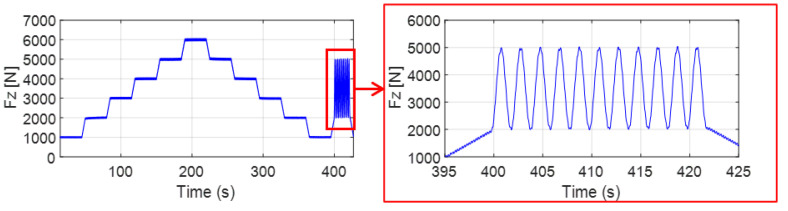
Vertical loading procedure.

The test was performed under controlled vertical load $F_{z}$, tire pressure, and speed conditions, as summarized in Table 3. The comparative analysis is based on test conditions with a vehicle speed of 30 km/h and a tire pressure of 260 kPa, and the vertical loading procedure is shown in Figure 7.

### 3.2. Raw Sensor Signal Comparison

**Figure 8:**
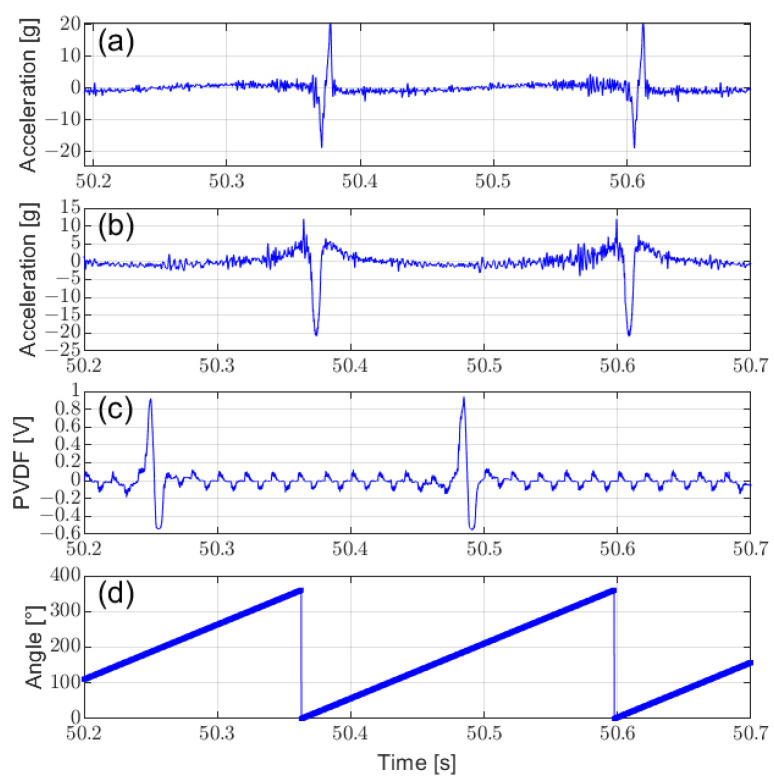
Comparison of raw signals from different tire sensor types: (**a**) circumferential acceleration, (**b**) radial acceleration, (**c**) PVDF sensor signals, and (**d**) corresponding rotation angle.

Figure 8 presents the characteristic response at the tire contact patch captured by the middle accelerometer and PVDF sensor through high-frequency sampling. The waveforms from both the accelerometer and PVDF sensor show a 180° phase difference, corresponding to their respective installation positions. Minimal changes were observed in the Y-axis acceleration, so it is omitted. The circumferential acceleration (Figure 8a) and radial acceleration (Figure 8b), along with the PVDF sensor signals (Figure 8c), exhibit distinct features, such as peak and valley characteristics, at the contact patch. These waveform characteristics align with the finite element simulation results shown in Figure 3a, Figure 3c, and Figure 4c, respectively, confirming the accuracy of the FEM simulation. This alignment indicates that these features can effectively reflect tire–ground interaction. By extracting these feature points and integrating them with the angular data (Figure 8d), the contact length is calculated.

### 3.3. Comparison of Sensor Signals at Different Tire Positions

**Figure 9:**
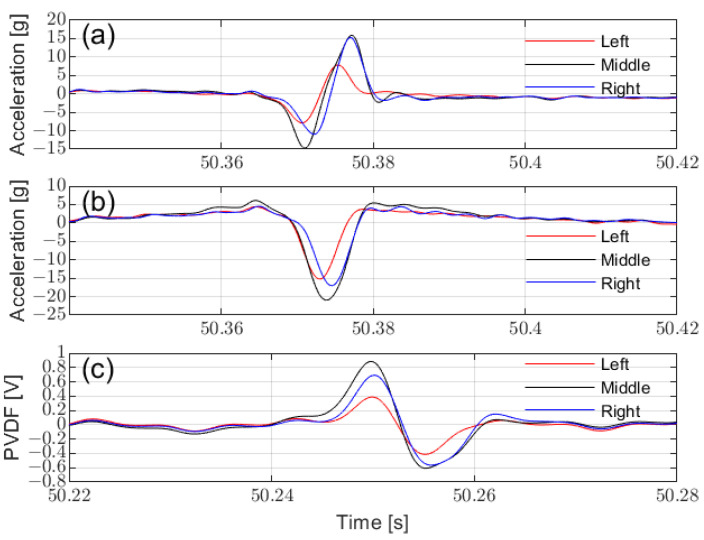
Comparison of tire sensors at different installation positions: (**a**) circumferential acceleration, (**b**) radial acceleration, and (**c**) PVDF signal.

**Figure 10:**
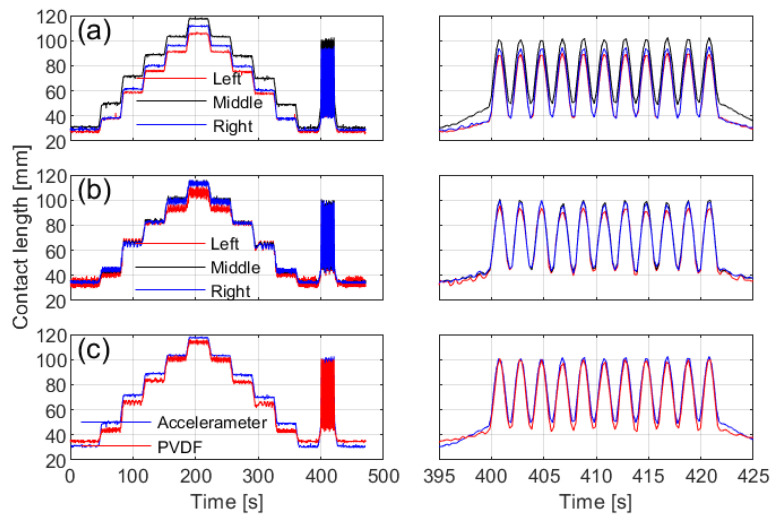
Comparison of contact lengths obtained from different installation positions and sensor types. The left side shows the full-duration curves, while the right side displays the dynamic test segment: (**a**) from circumferential acceleration, (**b**) from the PVDF sensor, and (**c**) from the middle sensor among the two sensor types.

Figure 9 compares the raw characteristic signals from sensors of the same type mounted at different positions. The sensor mounted at the middle produces the strongest signal and exhibits the longest characteristic duration, corresponding to a greater calculated contact length (Figure 10). This aligns with the FEM simulation results and demonstrates that placing multiple sensors at different locations within the tire allows for a more comprehensive capture of tire–ground contact characteristics, enabling the prediction of additional relevant information, especially under lateral forces and other mixed conditions. Since the signal from the middle position is easier to identify (due to its higher amplitude and longer corresponding contact length), the sensor at the middle can be used to calculate contact length and predict vertical load.

### 3.4. Comparison of Contact Length Calculation Methods

Figure 10 compares the contact lengths calculated from signals recorded at different installation positions and sensor types. The tire contact length, *L*, is calculated using the circumferential acceleration and PVDF signals as follows:

(1) $$L=2R\sin\left(\frac{\pi \theta }{90}\right),$$

where *R* is the rolling radius and θ represents the angular difference between the feature points, determined from the valleys and peaks of the circumferential acceleration and PVDF signals.

The contact lengths calculated from circumferential acceleration (Figure 10a) demonstrate noticeable differences across installation positions, with the middle sensor capturing the longest length, consistent with the tire’s contact patch shape. In contrast, measurements from the PVDF sensor (Figure 10b) show greater fluctuations, though the middle position still records the longest length.

Figure 10c presents a comparison of the contact lengths measured by different sensor types at the middle position. The PVDF sensor data exhibit higher variability, whereas the measurements from both methods reveal only minor overall differences.

### 3.5. Comparison of Sensor Stability

**Figure 11:**
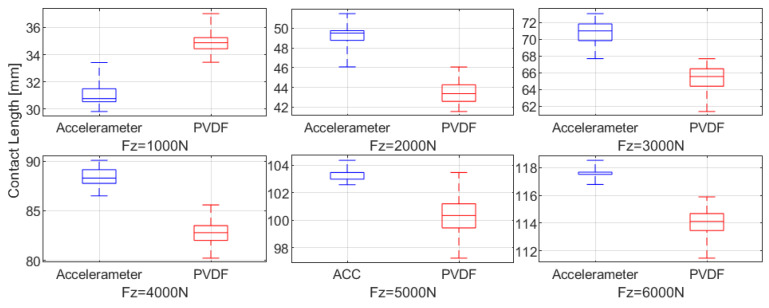
Comparison of contact length distribution at different loads using different sensor types: accelerometer versus PVDF (box plot representation).

**Figure 12:**
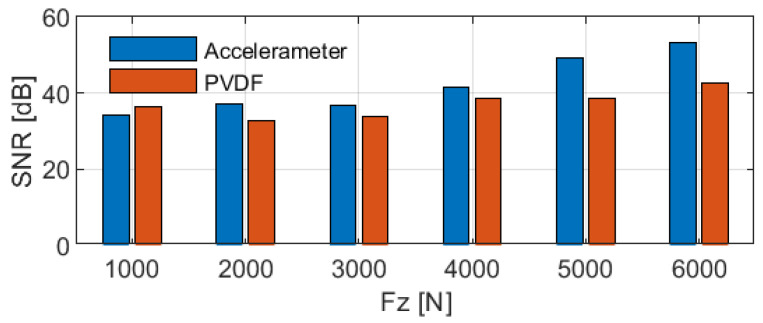
Comparison of contact length SNR using different sensor types: accelerometer versus PVDF.

To further compare the prediction performance of the two sensor types for ground contact characteristics, a box plot of the contact length predictions under steady vertical load conditions ranging from 1000 N to 6000 N is shown in Figure 11, based on the data from Figure 10c. Additionally, the signal-to-noise ratio (SNR) is calculated, with the SNR for each vertical load condition presented in Figure 12 and the mean SNR in Table 4. The SNR is calculated using the formula:

(2) $$SNR=10\times \log_{10}\left(\frac{{\mathrm{mean}}^{2}}{{\mathrm{std}}^{2}}\right)$$

where mean represents the mean of the signal and std represents the standard deviation of the signal, with the SNR measured in decibels (dB).

Except at low loads (Fz = 1000 N), the PVDF sensor exhibits greater fluctuations compared to the accelerometer, as evidenced by the wider interquartile range, larger spread in the box plot, and lower SNR at higher loads. These observations indicate that the PVDF sensor offers lower stability in tire contact characteristic estimation.

### 3.6. Vertical Load Estimation Performance Comparison

**Figure 13:**
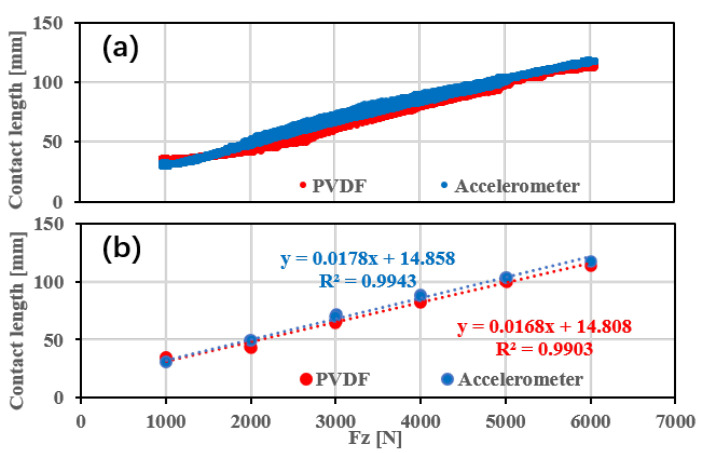
Comparative analysis of vertical load estimation performance using different sensor types: (**a**) complete dataset with dynamic loading and (**b**) average steady-state data with linear regression.

Figure 13 examines the relationship between contact length and vertical load based on different sensor types, with driving speed and tire pressure held constant. Both accelerometer and PVDF sensor measurements exhibit a linear trend, where contact length increases as vertical load rises. The accelerometer consistently records slightly greater contact lengths compared to the PVDF sensor, except at low vertical loads (below 1500 N).

Figure 13b shows the average contact lengths during the steady load phase. Both the accelerometer and PVDF sensor demonstrate a strong linear correlation between the calculated contact lengths and vertical load, with the coefficients of determination ($R^{2}$) provided in Table 4. The accelerometer exhibits a higher $R^{2}$, indicating better linearity compared to the PVDF sensor.

In summary, as indicated by the parameters in Table 4, the accelerometer demonstrates better stability (characterized by the mean SNR of the obtained contact length) and linearity (characterized by the $R^{2}$ value from the linear fit in vertical load prediction) compared to the PVDF sensor.

### 3.7. Vertical Load Estimation Under Multi-Factor Conditions

The comparative analysis results demonstrate that the accelerometer shows a clear advantage over the PVDF sensor in vertical load estimation. The contact length predicted by the accelerometer is more stable, and its linear relationship with vertical load is stronger. Therefore, the subsequent analysis uses circumferential acceleration to estimate the vertical load.

**Figure 14:**
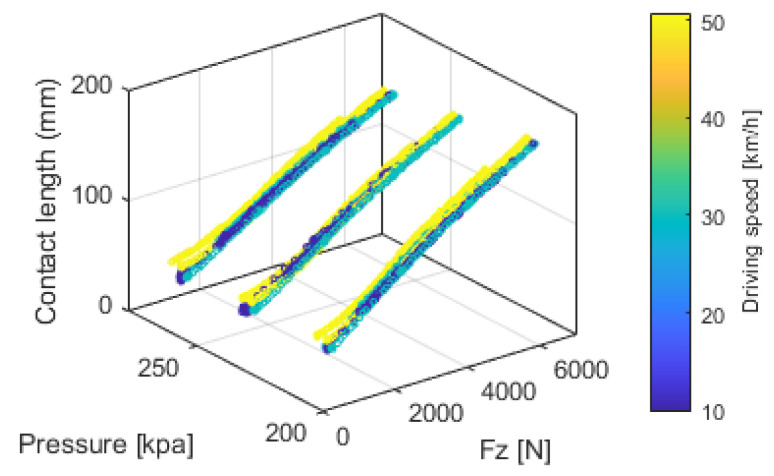
Relationship among contact length, vertical load Fz, tire pressure, and driving speed.

Similar vertical loading experiments were conducted under varying tire pressures and vehicle speeds. Figure 14 shows the results derived from the circumferential acceleration of the middle-mounted accelerometer. The analysis indicates that the vertical load has a dominant effect on the contact length, while tire pressure and speed exert comparatively smaller but still nonnegligible influences on the contact patch length.

**Figure 15:**
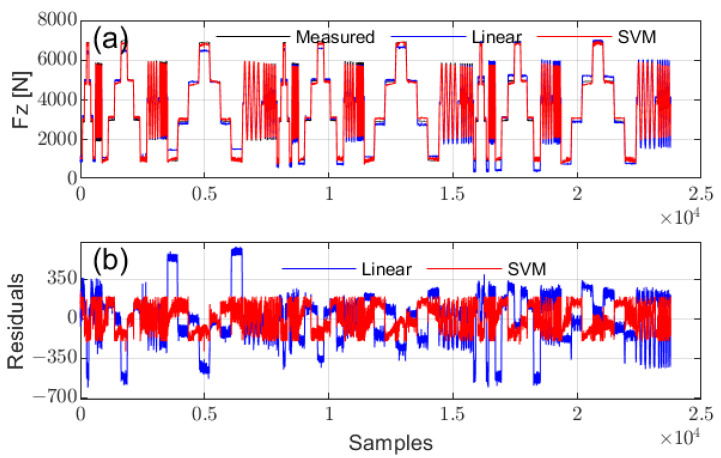
Prediction performance of vertical load $F_{z}$ using linear fit and SVM: (**a**) comparison of predictions, (**b**) prediction residuals.

Vertical load $F_{z}$ can be estimated based on contact length under specific tire pressures and vehicle speeds. Both linear regression and SVM regression [38] were applied, using contact length, tire pressure, and speed as input variables. The SVM regression employed a Gaussian Radial Basis Function (RBF) kernel, with automatic kernel scaling and standardized inputs to model the nonlinear relationship between variables. Figure 15 illustrates the predicted $F_{z}$ values and their corresponding errors. The mean absolute percentage errors (MAPE) for linear regression and SVM regression were 8.7771% and 3.6987%, respectively, demonstrating the superior accuracy of SVM. Nevertheless, linear regression remains a practical option due to its computational simplicity.

### 3.8. Experimental Summary

Experimental findings confirmed that both in-tire accelerometer and PVDF sensor characteristics aligned with simulation results, validating their use for vertical load estimation. Sensor placement at various locations provided comprehensive data, with middle-positioned sensors offering the most accurate vertical load predictions. However, for vertical load prediction, sensors positioned at the tire’s middle yielded the most accurate results. Furthermore, the comparison of sensor types indicated that the contact length derived from accelerometer signals exhibited greater stability and a stronger linear correlation with vertical load than the PVDF sensor. Consequently, the subsequent road tests will primarily utilize the accelerometer-based method for vertical load estimation.

## 4. Implementation and Road Validation

Building on the findings from the bench tests, this section focuses on the implementation of the vertical load estimation method for real-world driving scenarios. Considering the superior characteristics of accelerometers and the computational efficiency of linear regression, the proposed method employs an accelerometer-based sensing approach combined with a linear fitting model for vertical load prediction. To validate its performance under actual road conditions, two experimental setups were developed. First, a high-performance road testing system was implemented, leveraging high-precision in-tire sensors and the NI CompactRIO data acquisition platform. This setup ensures high sampling rates, synchronization accuracy, and reliable real-time computation.

Additionally, a cost-effective, product-level solution, the ITTU, was deployed for on-road testing. The ITTU features a compact hardware architecture, wireless RF transmission, and battery-powered operation. Although its performance is slightly lower than that of the high-performance setup, it offers significant cost savings, making it more suitable for practical deployment and commercialization.

Both testing setups were integrated into the intelligent tire development and testing platform developed by our research group, which is further detailed in our other publications.

### 4.1. High-Performance Testing System for Road Validation

**Figure 16:**
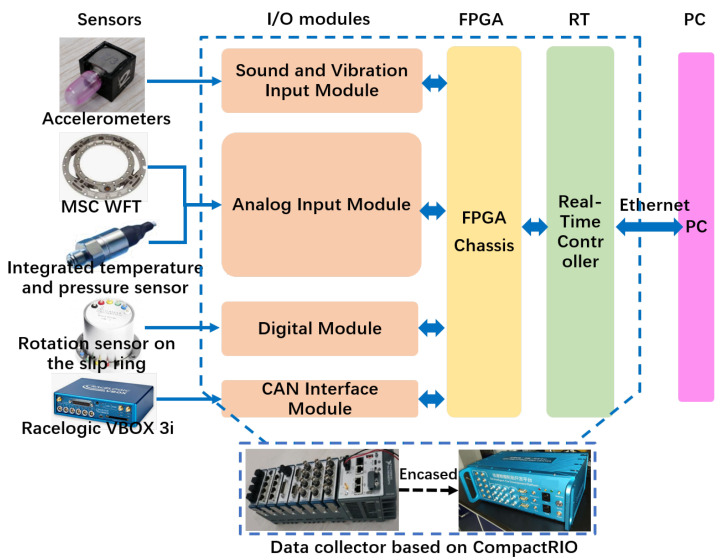
Experimental setup and hardware architecture for the road test.

**Figure 17:**
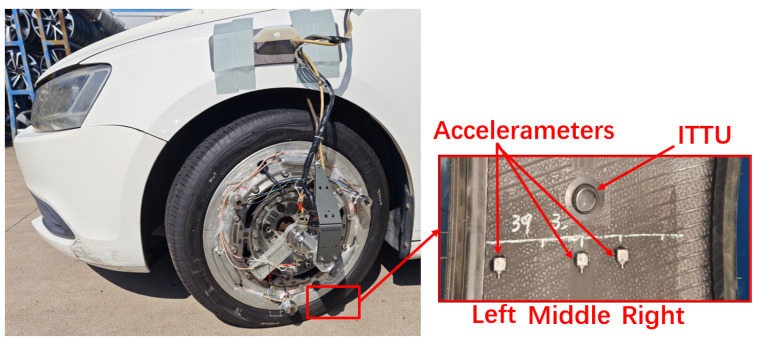
Road test images.

Figure 16 presents the overall architecture of the high-performance vertical load estimation system for road testing, while Figure 17 shows the experimental equipment and testing setup.

Three triaxial accelerometers were asymmetrically mounted inside the tire, including one at the middle, to capture ground contact characteristics across different installation positions. The WFT system (Michigan Scientific Corporation, Charlevoix, MI, USA) was employed to measure six-dimensional wheel forces and moments, serving as a reference for evaluation and calibration. Additionally, the Racelogic VBOX 3i (Racelogic Ltd., Buckingham, UK) recorded vehicle motion data, providing driving condition information as a reference for test scenarios. A slip ring, integrated with a rotational sensor, was used to maintain a stable wired connection during data acquisition while also providing precise tire rotation angle measurements. The data acquisition and processing unit was built on the NI CompactRIO platform, featuring a modular and distributed architecture consisting of I/O modules, a Field Programmable Gate Array (FPGA) chassis, a Real-Time controller (RT), and a host personal computer (PC). This system enables high-frequency synchronized sampling at 50 kHz for tire sensors, ensuring real-time performance, precise synchronization, and high computational efficiency with optimized data storage capabilities.

### 4.2. Deployment of the Intelligent Tire Test Unit

**Figure 18:**
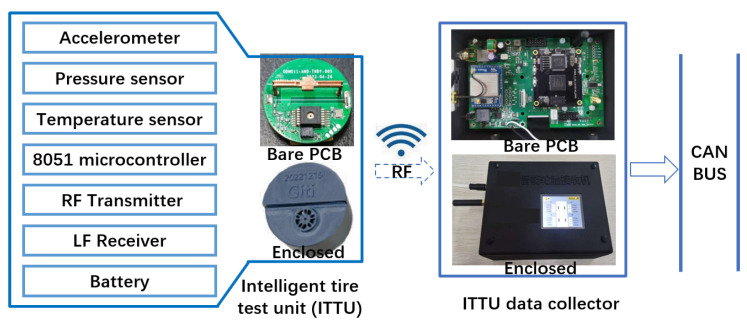
Overall system architecture of the Intelligent Tire Test Unit (ITTU).

Figure 18 illustrates the system architecture and a photo of the self-developed ITTU module. It integrates a single-axis accelerometer, pressure sensor, and temperature sensor, all managed by an 8051 microcontroller and powered by an internal battery. The ITTU wirelessly transmits data via RF to a centralized receiver, capable of handling multiple units. Processed data are transmitted through a Controller Area Network (CAN) bus for seamless integration with vehicle systems and analysis platforms.

Vertical load estimation uses linear regression based on the radial acceleration derivative, considering its simplification of calculations and computational efficiency for product-level deployment.

### 4.3. Experimental Conditions and Data Acquisition

**Figure 19:**
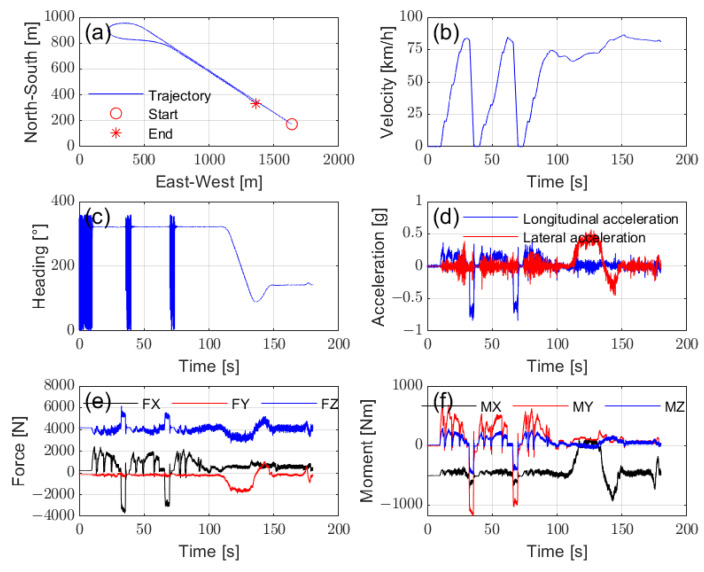
Data acquisition of the test conditions: (**a**) trajectory, (**b**) vehicle velocity, (**c**) heading angle, (**d**) vehicle acceleration, (**e**) wheel forces, and (**f**) wheel moments.

**Figure 20:**
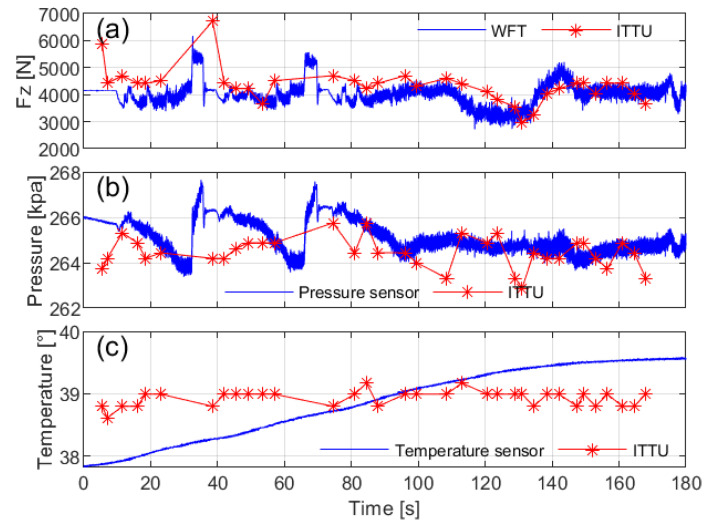
Validation of ITTU sensing performance compared to high-performance sensors: (**a**) vertical load $F_{z}$, (**b**) tire pressure, and (**c**) tire temperature.

Since the feasibility of stable driving conditions, such as constant-speed straight-line travel, has been demonstrated through bench testing, the focus of road test data analysis shifts to examining various dynamic driving conditions. Figure 19 illustrates the test conditions, which consist of two phases of acceleration and deceleration, followed by a turn and straight-line travel. Figure 19a–d present vehicle motion data from the VBOX, including the vehicle’s trajectory, velocity, heading angle, and acceleration, offering detailed information on the driving behavior during the test. Figure 19e,f show wheel forces and moments measured by the WFT system, highlighting a significant increase in vertical load during braking. These measurements serve as reference signals for the prediction and evaluation of vertical load. Additionally, Figure 20b,c provide in-tire pressure and temperature data with high-precision, high-frequency sampling.

### 4.4. Validation of Vertical Load Estimation

**Figure 21:**
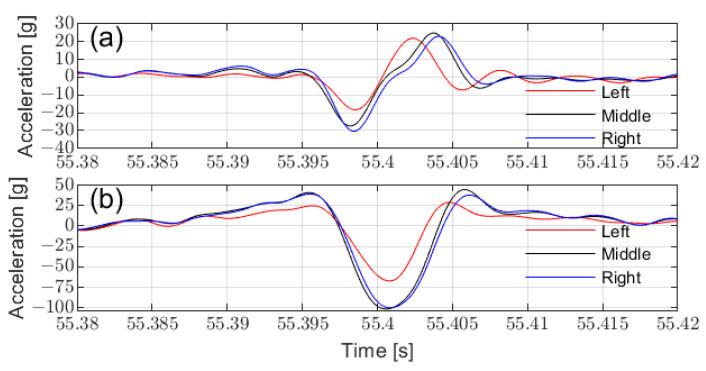
Acceleration characteristics at different installation positions: (**a**) circumferential acceleration, (**b**) radial acceleration.

Figure 21 presents the characteristics of in-tire accelerometer signals during road driving. Accelerometers positioned farther from the middle recorded shorter signal durations and lower amplitudes, consistent with FEA and bench test findings.

**Figure 22:**
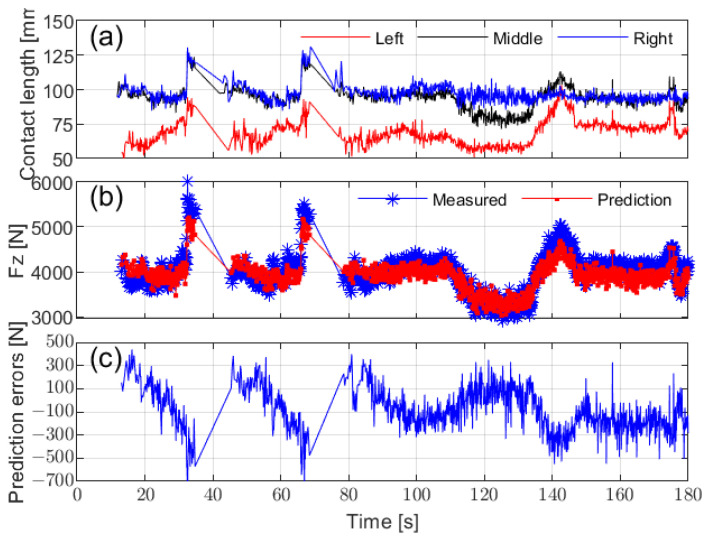
Contact length calculation and vertical load estimation: (**a**) calculated contact length based on circumferential acceleration from different installation positions, (**b**) vertical load estimation based on the middle contact length, and (**c**) vertical load estimation residuals.

Figure 22a illustrates the contact lengths calculated from circumferential acceleration at different installation positions across various driving conditions. The left-side accelerometer recorded the shortest ground contact length, while the right-side accelerometer exhibited reduced sensitivity during turns. This variation in contact lengths due to sensor installation positions and driving conditions highlights the importance of sensor placement when predicting vertical load. To minimize prediction errors, it is optimal to install the sensor in the middle of the tire.

Additionally, by placing the prediction during stable tire operation, such as avoiding prediction during turns, the influence of sensor placement on vertical load prediction can be further minimized. Figure 22b,c compare the predicted vertical load, derived using a linear regression method based on the middle contact length, with the actual vertical load measured by the WFT system. The results confirm the effectiveness of the prediction method under real-road conditions.

### 4.5. ITTU Performance Evaluation and Comparative Analysis

The instrumented front-left (FL) tire with both high-performance sensors and ITTU enables the calibration and validation of ITTU performance, as shown in Figure 20. While the ITTU generally provides accurate data, some delays and reduced sensitivity to rapid changes due to low sampling frequency were observed. However, the system demonstrated reliable performance during steady-state driving, such as constant-speed, and straight-line scenarios.

**Figure 23:**
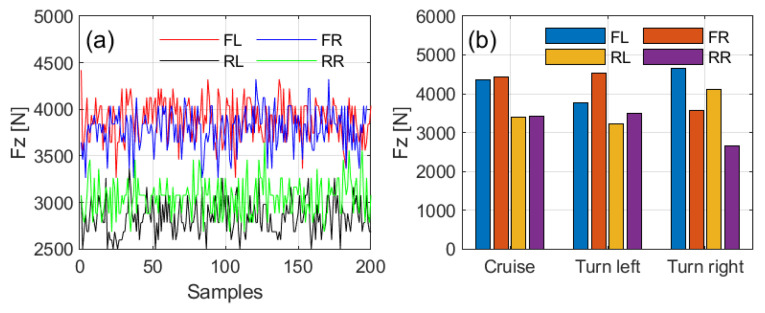
Vertical load estimation results of ITTU for all four tires under driving conditions: (**a**) real-time data during a constant-speed drive at 45 km/h, (**b**) average vertical load during cruising and turning.

Additionally, the four ITTUs, one on each tire, provide complete tire information. Figure 23a shows the vertical load of all four tires during a constant-speed drive at 45 km/h, demonstrating consistently stable vertical loads for each tire under this condition. Figure 23b compares the average vertical load during cruise and turning, highlighting load transfer among the tires and confirming the reliability of the vertical load estimation implemented in the ITTU.

## 5. Conclusions

This study provides a comprehensive investigation into vertical load estimation using intelligent tire technology, offering a comparative analysis of accelerometer-based and strain-based sensing methods. The research spans the entire development cycle, from theoretical modeling and bench testing to real-world validation and product-level evaluation.

Finite element analysis (FEM) was conducted to evaluate the acceleration and strain characteristics at various tire positions, confirming the feasibility of both sensor types for vertical load prediction. High-precision bench tests revealed that accelerometers offer superior stability and linearity compared to PVDF sensors. Based on these findings, vertical load prediction algorithms were developed using accelerometer data, vehicle speed, and tire pressure. The algorithms, using both Support Vector Machine (SVM) and linear regression models, demonstrated good accuracy in predicting vertical load.

The proposed method was rigorously validated under real-road conditions using high-performance instruments across various driving scenarios, including constant speed, acceleration, braking, and cornering. Additionally, it was deployed in the self-developed ITTU for product-level validation, confirming the effectiveness of the method in real-world applications.

This work contributes to the practical deployment of intelligent tire technology by providing a robust framework for vertical load estimation. Future research should focus on further analyzing sensor characteristics to improve dynamic performance and exploring fusion methods to enhance sensor accuracy. Additionally, extending this methodology to predict other tire parameters, such as longitudinal and lateral forces, could further advance intelligent tire technology.

## Figures and Tables

**Table 1:** Yeoh trinomial parameter table of tire rubber.

Material Name	C10	C20	C30
Tread Rubber	0.728023	−0.26918	0.084875
Sidewall Rubber	0.484964	−0.13221	0.042146
Belt Layer Rubber	1.242562	−0.49497	0.212215
1# Belt Reinforcement Rubber	1.242562	−0.49497	0.212215
2# Belt Reinforcement Rubber	1.242562	−0.49497	0.212215
Shoulder Pad Rubber	1.242562	−0.49497	0.212215
Tire Body Rubber	0.874706	−0.22225	0.087177
Inner Liner Rubber	0.579786	−0.22229	8.55 $\times \;10^{-2}$
Triangle Rubber	3.180557	−5.39609	6.926711
Tread Wear Rubber	1.366444	−0.5121	0.212145
Steel Wire Hoop Rubber	3.180557	−5.39609	6.926711

**Table 2:** Material parameters of rebar.

Cord Type	Poisson’s Ratio	Young’s Modulus (MPa)	Cord Cross-Sectional Area (mm²)	Cord Spacing (mm)	Angle * (°)
Belt Layer 1	0.3	188,500	0.1717	1.25	112
Belt Layer 2	0.3	188,500	0.1717	1.25	68
Crown Layer	0.4	6948.339	0.302	1.25	45
Tire Body Cord	0.4	9597	0.302	0.274	0
Steel Wire Hoop	0.3	206,000	1.3273	1.7	90

* The angle is defined with the tire’s meridian as the reference, where 90° is the baseline.

**Table 3:** Experimental conditions.

Vertical Load (N)	Tire Pressure (kPa)	Test Speed (km/h)
1000, 2000, 3000, 4000, 5000, 6000, Dynamic Sinusoidal Loading	210, 240, 250	10, 30, 50

**Table 4:** Comparison of sensing performance across different sensor types.

Sensor Type	Stability (Mean *SNR*)	Linearity ($R^{2}$)
Accelerometer	41.9528	0.9943
PVDF	37.1686	0.9903

## Data Availability

Data are contained within the article.

## Abbreviations

The following abbreviations are used in this manuscript:

FEM	Finite element modeling
IEPE	Integrated Electronics Piezoelectric
PVDF	Polyvinylidene Fluoride
SVM	Support Vector Machine
ITTU	Intelligent Tire Test Unit
WFTs	Wheel force transducers
FEA	Finite element analysis
FPGA	Field Programmable Gate Array
RT	Real-Time Controller
PC	Personal computer
RBF	Radial Basis Function
MAPE	Mean absolute percentage error
CAN	Controller Area Network
SNR	Signal-to-noise ratio
